# Heteroepitaxial 3C-SiC for MEMS Applications

**DOI:** 10.3390/mi17040502

**Published:** 2026-04-21

**Authors:** Angela Garofalo, Annamaria Muoio, Luca Belsito, Sergio Sapienza, Matteo Ferri, Alberto Roncaglia, Francesco La Via

**Affiliations:** 1 Materials Science Department, Milano—Bicocca University, Via R. Cozzi 55, 20125 Milan, Italy; 2 CNR-IMM, Strada VIII, 5, 95121 Catania, Italy; annamaria.muoio@cnr.it; 3 CNR-ISMN Bologna Unit, Via Gobetti, 101, 40129 Bologna, Italy; luca.belsito@cnr.it (L.B.); sergio.sapienza@cnr.it (S.S.); matteo.ferri@cnr.it (M.F.); alberto.roncaglia@cnr.it (A.R.)

**Keywords:** silicon carbide, MEMS, COMSOL, isotropic loss factor, anisotropic loss factor, pressure sensor, mechanical resonator

## Abstract

Silicon carbide (SiC) has emerged as a highly attractive material for microelectromechanical systems (MEMS) operating in harsh environments, owing to its outstanding mechanical, thermal, and chemical properties. This review provides a comprehensive overview of the advantages and limitations of SiC-based MEMS, with particular emphasis on the strong interdependence between material structure, mechanical properties, and epitaxial growth processes. The role of defects, residual stress, and crystal quality is discussed in relation to device performance and reliability. Special attention is devoted to cubic SiC grown on silicon substrates, highlighting how growth-induced features influence the mechanical response of micromachined structures. Furthermore, a detailed analysis of the quality factor (Q-factor) is presented for 3C-SiC (111)/Si resonators, including the development of analytical models and their validation through numerical simulations performed using COMSOL Multiphysics (Version 6.1). The necessity of incorporating anisotropic loss factors in numerical modeling is demonstrated to be essential for accurately describing the experimentally observed behavior. This review aims to provide design guidelines and modeling strategies for the optimization of SiC MEMS, supporting their further development for high-performance and extreme-environment applications, including pressure sensors, mechanical resonators and high-stress-tolerant sensors.

## 1. Introduction

The term microelectromechanical systems (MEMS) refers to devices or arrays of devices whose characteristic dimensions range from a few micrometers to several millimeters. These systems comprise collections of microsensors capable of detecting and responding to environmental changes at the microscale while producing responses at the macroscale, thereby enabling the acquisition of information on material properties, geometry, and mechanical or electromagnetic effects [1].

At present, MEMS are widely employed in a broad range of devices, including thermal, pressure, mass and temperature sensors, resonators, pumps, motors, optical sensors, and transmission systems [2,3,4,5,6,7,8], as well as in microsurgical, medical, and biomedical devices [9]. In addition, they are increasingly utilized in geophysical monitoring applications, including earthquake [10,11,12] and volcanic eruption monitoring systems [13,14]. Several review articles have addressed SiC for MEMS and related microsystems. Cimalla et al. [2] provided a broad overview of group-III nitride and SiC-based MEMS, covering material properties and technology, while La Via et al. [14] recently surveyed emerging SiC applications beyond power electronics. Earlier reviews have also addressed the processing, integration, and reliability of SiC thin films [1]. However, no review has to date systematically examined the interplay between heteroepitaxial growth quality, mechanical properties, and dynamic dissipation modeling for 3C-SiC specifically, nor demonstrated the necessity of anisotropic loss-factor models for predicting Q-factor trends in heteroepitaxial resonators. The present review addresses this gap by: (i) providing a comprehensive quantitative overview of how growth-induced defects and residual stress jointly determine the mechanical response of 3C-SiC MEMS, (ii) presenting and contextualizing the analytical Q-factor models applicable to this material system across a wide thickness range, and (iii) demonstrating that anisotropic damping formulations are not a mathematical luxury but a physical necessity for accurate device simulation. Given the remarkable success of MEMS-based sensors across such a broad range of applications, their potential deployment in so-called harsh environments is a natural progression. In this context, MEMS must be capable of reliable operation and of maintaining accurate performance even under prolonged exposure to extreme conditions, and they should be suitable for integration into different applications without suffering damage or mechanical failure.

Silicon carbide (SiC) is a promising alternative to silicon in MEMS devices. The mechanical properties of SiC, in particular, have been extensively studied to enable their effective exploitation in a variety of applications. These include a large Young’s modulus, which allows the achievement of higher resonance frequencies compared with conventional materials, and a significant quality factor (Q-factor), which quantifies the energy dissipation within the system and directly influences the noise level and, consequently, the resolution of resonant sensors. A high resonator quality factor is essential for applications such as high-precision mass sensing. Moreover, optimal resonator performance is obtained when the product of the resonant frequency and the quality factor is maximized. In microbeam-based resonators, the resonant frequency is governed by the residual stress of the material, as well as by its elastic properties, dimensions, and geometric configuration.

However, several challenges remain for the full development of high-performance devices based on heteroepitaxial 3C-SiC, particularly with regard to material quality [15]. The significant defect density observed in 3C-SiC layers is primarily a consequence of the large mismatch in lattice parameters (approximately 19% at room temperature) and thermal expansion coefficients (about 23% at deposition temperatures and 8% at room temperature) between 3C-SiC and silicon [16]. Following growth, the stress generated during the cooling phase is tensile, leading to an upward (concave) bending of the wafer and, in many cases, to the formation of cracks within both the epitaxial layer and the silicon substrate. The magnitude of this deflection depends on several factors, including the crystal and substrate dimensions, the thicknesses of the epitaxial layer and the silicon substrate, and the crystallographic orientation of both the epitaxial film and the substrate [17]. Consequently, various approaches have been developed to mitigate defect density and reduce wafer bowing induced by residual stress. Residual stress is also influenced by growth conditions, particularly during the carbonization and chemical vapor deposition (CVD) steps; moreover, the effect of layer doping on stress has been reported [18,19], and the spatial distribution of defects within the epitaxial layer has been shown to play a significant role as well [20,21]. It should be noted that CVD-based heteroepitaxial growth on silicon is not the only route to SiC micro- and nanostructures. For bulk SiC polytypes (4H, 6H), and increasingly for thick 3C-SiC layers, precision machining technologies represent an important complementary fabrication approach. Ultra-precision and field-assisted cutting techniques, in which external energy fields (thermal, electrical, magnetic, or optical) are used to modify the deformation zone and facilitate material removal in brittle ceramics, have been demonstrated to produce SiC surfaces with nanometric finish and controlled subsurface damage [22]. These methods are particularly relevant when the starting material is a bulk SiC wafer or when the required feature sizes and surface quality cannot be achieved by wet or dry etching alone. At the nanoscale, the fundamental mechanisms of material removal in brittle solids, including the ductile-to-brittle transition and the role of dislocations and crack propagation in determining cutting forces and surface integrity, have been studied in detail in the context of nanometric cutting [23]. Understanding these mechanisms is important for the microfabrication of high-performance SiC MEMS components where surface roughness and subsurface damage directly affect mechanical losses (see Section 6). The present review focuses on CVD-grown heteroepitaxial 3C-SiC on Si, because this is the technology platform that enables wafer-scale integration and that introduces the unique combination of residual stress and crystallographic defects that are central to the modeling sections. Bulk SiC machining, while important and rapidly evolving, falls outside the scope of this work. This paper provides a comprehensive overview of the advantages and disadvantages of SiC MEMS technology. In particular, the results clearly indicate the connection between the material structure, mechanical properties and growth process. Furthermore, a detailed analytical model of the Q-factor in the case of 3C-SiC (111)/Si and numerical simulations performed with COMSOL Multiphysics (Version 6.1) are presented, highlighting the need to account for anisotropic loss factors. Finally, the relevance of SiC MEMS applications is discussed, with particular emphasis on pressure sensors, mechanical resonators and devices operating in harsh environments, where the superior thermal stability, mechanical robustness and chemical inertness of silicon carbide enable performance beyond that of conventional Si-based MEMS.

## 2. Advantages of SiC for MEMS

### 2.1. High Frequency and Stiffness

3C-SiC is a material of choice for high-performance and harsh-environment MEMS due to its intrinsic properties that significantly surpass those of conventional silicon; SiC possesses a remarkably large Young’s modulus. Measurements on epitaxial 3C-SiC films on silicon substrates determined an average in-plane Young’s modulus ranging between 211 and more than 500 GPa (Table 1). The hardness also shows a large spread, ranging from 31 to 700 GPa (Table 1). This large range of values strongly depends on the deposition processes, the layer thickness, and their crystallographic orientations. In a more recent work, with several wafers grown on Si (100) (Table 2), it was observed that both the Young modulus and the residual stress depend on the film thickness and the growth rate, which strongly influences the defect density of the layer [2,24].

Among the various SiC polytypes, this review focuses specifically on cubic 3C-SiC for three interconnected reasons. First, 3C-SiC is the only SiC polytype that can be grown hetero epitaxially on large-area (150 and 200 mm) silicon substrates by standard chemical vapor deposition, making it uniquely compatible with the existing CMOS fabrication infrastructure. This compatibility enables cost-effective wafer-scale MEMS production, a critical advantage for commercial deployment, which is not available to bulk 4H-SiC or 6H-SiC, which require dedicated bulk micromachining on expensive SiC wafers. Second, the large lattice and thermal mismatch between 3C-SiC and Si generates a substantial built-in tensile residual stress ($\sigma_{0}$ typically in the range 100–1000 MPa) that, rather than being a mere fabrication defect, is a functional resource: this prestress increases the resonance frequency and, through the dissipation dilution mechanism [41,42], can boost the effective quality factor by one to two orders of magnitude above the intrinsic material limit. Exploiting this mechanism requires, however, a quantitative understanding of how stress, defect density, and film thickness jointly control Q, exactly what this review provides. Third, the specific defect structure of heteroepitaxial 3C-SiC, dominated by stacking faults and partial dislocations lying on 111 planes, introduces a crystallographical anisotropic perturbation of the stiffness tensor that is absent in bulk polytypes and in isotropic polycrystalline films. This anisotropy manifests directly in the directional dependence of energy dissipation and necessitates the Voigt-based anisotropic damping formulation discussed in Section 7. In this sense, 3C-SiC is not only a technologically important material but also a uniquely instructive model system for studying the coupling between epitaxial growth, crystallographic symmetry, and mechanical dissipation at the microscale.

The heteroepitaxial growth of 3C-SiC on Si is significantly hindered by the large mismatch in lattice parameters (approximately 20%) and thermal expansion coefficients (approximately 8%) between the two materials. This substantial mismatch is responsible for the formation of an elevated density of structural defects at the interface, including misfit dislocations, twins, and stacking faults (SFs) [43,44,45,46]. The presence of such defects within the film perturbs the crystalline structure of the system and may lead to significant modifications of the elastic properties of the material. A commonly reported feature of the 3C-SiC/Si system is that extended defects generated at the 3C-SiC/Si interface, such as stacking faults (SFs) and micro-twins (μ-twins), tend to mutually annihilate during epitaxial growth, leading to a progressive reduction in their density with increasing distance from the interface. This decrease in defect density is evidenced by the narrowing of the full width at half maximum (FWHM) of the rocking curves associated with the 3C-SiC X-ray diffraction peaks as the epilayer thickness increases. Further confirmation of the improvement in crystalline quality with increasing film thickness is provided by the reduction in the FWHM of the Raman 3C-SiC transverse optical (TO) mode. Several studies have reported an increase in Young’s modulus of 3C-SiC films with increasing epilayer thickness [47,48]. This behavior has been reasonably attributed to the progressive reduction in the density of extended defects and the consequent enhancement of the structural quality of the 3C-SiC epilayer.

**Figure 1:**
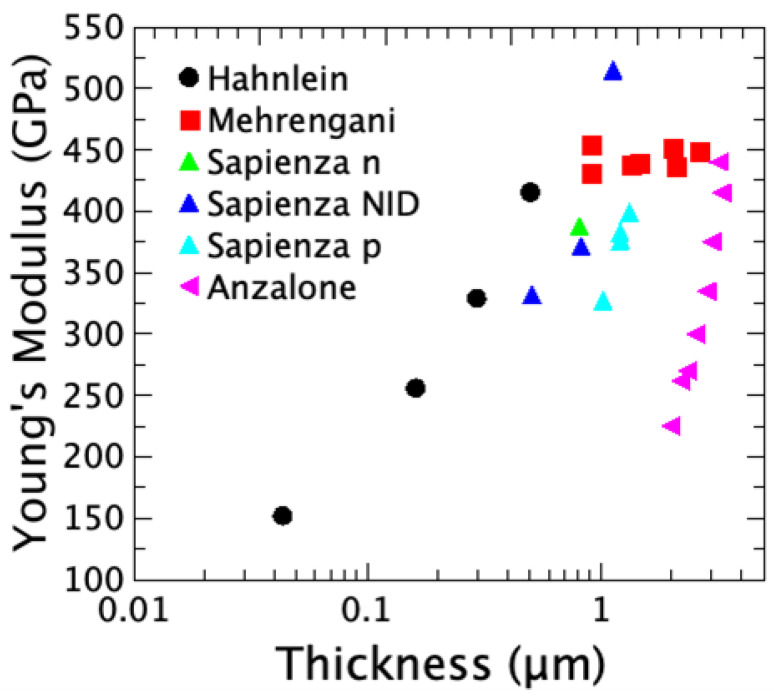
Comparison of measured values of Young’s modulus with other results from the literature about 3C-SiC grown on <100> silicon as a function of thickness. Black circle: [53]. Red Square: [54]. Green, blue, light-blue triangle: [55]. Purple triangle: [47].

**Figure 2:**
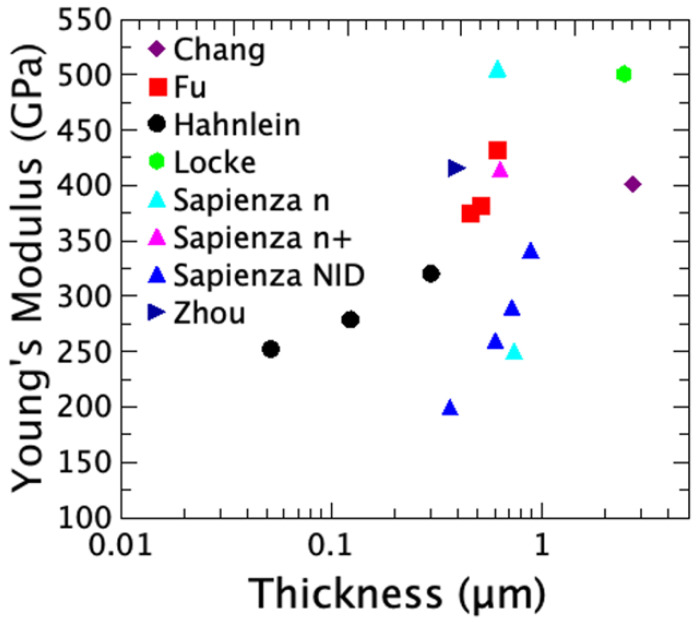
Comparison of measured values of E with other results from the literature about 3C-SiC grown on <111> silicon. Purple diamond: [56]. Red square: [57]. Black circle: [58]. Green circle: [25]. Light-blue, pink, blue triangle: [55]. Dark-blue triangle: [59].

As can be seen in the comparison reported in Figure 1 and Figure 2, the Young’s modulus increases, increasing the thickness of the layer. The difference in Young’s modulus for a fixed thickness in the different experiments reported in the figures is probably due to the different processes that can produce different defect densities. On the samples grown on <111> Si the n-type doping produces an increase in Young’s modulus, while in the material grown on <100> Si, the p-type doping produces a reduction in Young’s modulus. The increase in Young’s modulus, in 3C-SiC n-type or p-type doped, is likely related to the reduction in the SF density observed in this type of material in previous papers [49,50]. In those papers the increase in the annihilation of the SFs was measured on bulk samples, but a similar behavior has been expected even in thinner samples. These values of E/ρ enable the fabrication of microresonators with substantially higher intrinsic resonance frequencies compared to geometrically equivalent devices made of silicon or GaAs [51]. Specifically, the effective wave velocity $v={\left(E/\rho \right)}^{1/2}$, which governs the frequency in the bending-dominated regime, is approximately $1.5\times 10^{4}$ m/s for 3C-SiC, compared to $7.5\times 10^{3}$ m/s for Si and $4\times 10^{3}$ m/s for GaAs. It should be noted that the actual resonance frequency of a fabricated device depends not only on E/ρ but also on the beam geometry (length L, thickness h, width w), the residual stress state $\sigma_{0}$, and the vibration mode shape, as expressed by Equation (1). An elevated E/ρ ratio is, therefore, a necessary but not sufficient condition for high-frequency operation. The fundamental resonance frequency, *f*, of a doubly clamped beam of length *L*, and thickness, *t*, varies linearly with the geometric factor $t/L^{2}$, according to the simple relation:

(1) $$f=1.03\sqrt{\frac{E}{\rho }}\frac{t}{L^{2}},$$

where E is Young’s modulus and ρ is the mass density [52]. Further correction terms of an order higher than $t/L^{2}$ are expected to appear if the beams are under significant tensile or compressive stress.

### 2.2. High-Temperature and Harsh-Environment Operation

3C-SiC/Si MEMS devices have been demonstrated to operate reliably at temperatures above 300 °C, with potential operational limits reaching 700 °C (Table 3). This property is extremely important for all the applications where high-temperature operation is essential.

### 2.3. Exceptional Quality Factor (Q-Factor)

The high tensile residual stress inherent in heteroepitaxial SiC has been leveraged to create microstring resonators that achieve Q-factors over one million (typically in the $10^{5}$–$10^{6}$ range) [63] (Table 4). This exceptionally high Q-factor is crucial for reducing noise and maximizing sensitivity in resonant sensors, such as strain gauges. These exceptional Q-values were obtained under specific conditions: all measurements were performed in a high-vacuum (pressure below $10^{-6}$ mbar) to eliminate viscous air damping, and the resonators used were highly stressed nano string geometries (tensile stress $\sigma_{0}\geq$ 750 MPa, length/thickness aspect ratio L/h > 3000) designed to maximize the dissipation dilution factor D(h). Geometrically different 3C-SiC structures, or devices operating at lower stress or in non-vacuum environments, will exhibit substantially lower Q-values. The quality factor is therefore a device-level quantity that depends on geometry, stress, and environment, not solely on the material.

### 2.4. Comparative Overview of SiC Polytypes for MEMS Applications

Although this review focuses on heteroepitaxial 3C-SiC, a brief comparison with other SiC material forms is useful to contextualize the choices made in practice. The four main material platforms for SiC MEMS are: single-crystal 3C-SiC grown heteroepitaxially on Si, single-crystal 4H-SiC and 6H-SiC produced by sublimation from bulk wafers, and polycrystalline SiC (poly-SiC) deposited by CVD. 3C-SiC on Si is the only polytype compatible with large-area (100 and 150 mm) silicon wafer processing, making it uniquely scalable at low cost. However, the large lattice and thermal mismatch with Si generate a large density of extended defects (stacking faults, threading dislocations) that degrade the effective Young’s modulus and introduce residual stress gradients. These defects decay with increasing film thickness, so that material quality improves progressively but at the cost of increased thermal budget and film thickness. 4H-SiC and 6H-SiC are available as bulk single-crystal wafers and offer superior crystalline quality, wider bandgap, and higher thermal conductivity. However, they require dedicated bulk micromachining techniques (deep RIE, laser ablation, KOH etching after wafer bonding), are not directly compatible with Si CMOS fabrication, and are substantially more expensive. These polytypes are preferred for high-temperature piezoresistive pressure sensors and power MEMS but are less suited to wafer-scale resonator arrays. Poly-SiC can be deposited at lower temperatures and on arbitrary substrates, offering flexibility but at the cost of lower Young’s modulus (due to grain boundaries), higher intrinsic mechanical losses, and greater process variability. It is often used in composite resonator structures (e.g., Poly-Si/SiC) to engineer residual stress. The key trade-off for MEMS design can be summarized as follows: 3C-SiC on Si offers scalability and a large built-in tensile stress (favorable for Q through dissipation dilution) but requires careful management of defect density and stress gradient; bulk 4H/6H-SiC offers superior material quality but poor Si-process compatibility; poly-SiC offers process flexibility but inferior Q and stiffness.

## 3. Disadvantages of SiC for MEMS

The primary challenge for widespread adoption of 3C-SiC in MEMS lies in the difficulties associated with heteroepitaxial growth on silicon, particularly concerning defect management and residual stress.

### 3.1. High Density of Extended Defects

The lattice mismatch generates a significant density of crystallographic defects, especially stacking faults (SFs) and threading dislocations (TDs). These defects are concentrated at the interface and propagate toward the film surface. These defects strongly influence the mechanical properties of the MEMS devices (Section 3.3) and lead to a significant dependence of these properties on the thickness of the deposited film. Furthermore these defects produce a large residual stress as will be explained in the next section.

### 3.2. High and Inherent Residual Stress

In a thin-film/thick-substrate system, as is the case for the heteroepitaxy between 3C-SiC and Si, the residual strain field ($\epsilon_{res}$) results from two different contributions. Ex situ stress measurements, performed after the growth process, allow the determination of residual strain $\epsilon_{res}$ of the film, which is the sum of two contributions:

(2) $$\epsilon_{res}=\epsilon_{th}+\epsilon_{int}.$$

The first contribution is the thermoelastic strain $\epsilon_{th}$ caused by the difference in thermal expansion coefficients (α) between the film and the substrate and induced during cooling after growth. A thermally derived stress is set up when a film is deposited onto a heated substrate and then cooled to room temperature. The amount of contraction that each portion of the couple attempts to undergo is different due to the difference in their respective expansion coefficients. For situations where the film is much thinner than the substrate, and well bonded to the substrate, the stress related to the strain in the film at a temperature T is given by:

(3) $$\sigma_{th}=\frac{E_{f}}{1-\nu_{f}}\left(\alpha_{f}-\alpha_{s}\right)\left(T_{dep}-T\right),$$

where $E_{f}$, $\nu_{f}$, and $\alpha_{f}$ are Young’s modulus, Poisson’s ratio, and the thermal expansion coefficient of the film, respectively, while $\alpha_{s}$ is the thermal expansion coefficient of the substrate, and $T_{dep}$ is the deposition temperature. Since $\alpha_{SiC}>\alpha_{Si}$, the thermoelastic strain in any 3C-SiC film on Si is always of tensile nature ($\epsilon_{res}>0$). Each component of the film-substrate system is independent of any growth parameters, except temperature. The second term of the expression is closely related to the heteroepitaxy itself: the intrinsic contribution, $\epsilon_{int}$; it is the in situ strain at high temperature. It is mainly due to the lattice mismatch between the two different materials that, in the case of 3C-SiC and Si, about 20%, and starts manifesting itself from the early stage of growth. The lattice mismatch is accommodated by the distortion of the unit cells in the overlayer, resulting in a biaxially strained structure with a coherent interface. If the strain is incorporated into the epitaxial crystal coherently, the lattice constant of the epitaxial layer in the direction parallel to the interface is forced to be equal to the lattice constant of the substrate. The lattice constant of the epitaxial layer perpendicular to the substrate will be changed by the Poisson effect. If the parallel lattice constant is forced to shrink, or a compressive strain is applied, the perpendicular lattice constant will increase. Conversely, if the parallel lattice constant of the epitaxial layer is forced to expand under tensile strain, the perpendicular lattice constant will shrink. This type of coherently strained crystal is called pseudomorphic. The heteroepitaxy of 3C-SiC on Si introduces an intrinsic tensile residual stress due to the large lattice mismatch (about 20%) and thermal mismatch between the two materials [20]. In thin films, the overall residual stress consists of both uniform and gradient components. The uniform component is primarily associated with the substrate and induces a boundary-related linear pre-bending. Conversely, the gradient component leads to cantilever bending, producing a deflection whose orientation (positive or negative) is closely linked to the defect density within the epitaxial film. For the first approximation, the residual stresses can be represented using a linear equation as

(4) $$\sigma_{T}\left(z\right)\approx \sigma_{0}+\sigma_{1}\left(\frac{z}{h/2}\right).$$

**Figure 3:**
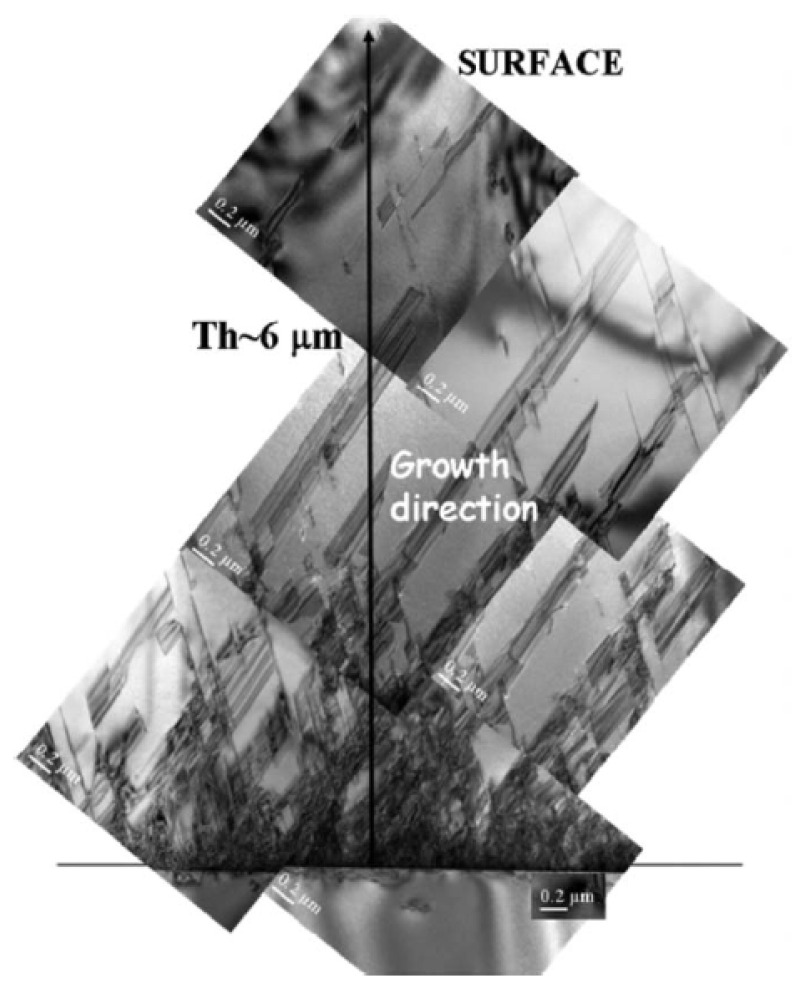
Cross-section TEM image showing (111) 3C-SiC from the interface with the Si to the surface. The black contrast close to the interface is due to the large density of defects generated by the heteroepitaxy. These defects become mainly stacking faults, shown as tilted lines or platelets, approaching the film surface [64].

The sequence of cross-sectional TEM images in Figure 3 illustrates the typical evolution of defects in 3C-SiC grown on Si as a function of film thickness (up to 6 µm). As shown in the figure, a large density of defects is observed near the film–substrate interface, appearing as a dark contrast region, while the defect density significantly decreases with increasing film thickness. This behavior highlights the limitations of the linear stress approximation. Micromachined Raman spectroscopy is a nondestructive technique capable of evaluating the stress distribution within epitaxial layers. Through a combined analysis, it is possible to assess the state of the epitaxial film by revealing the stress evolution from the silicon substrate interface toward the film surface. In particular, analysis of the transverse optical (TO) phonon mode enables probing both the crystalline quality, via the full width at half maximum (FWHM), and the residual stress, inferred from the peak position shift.

**Figure 4:**
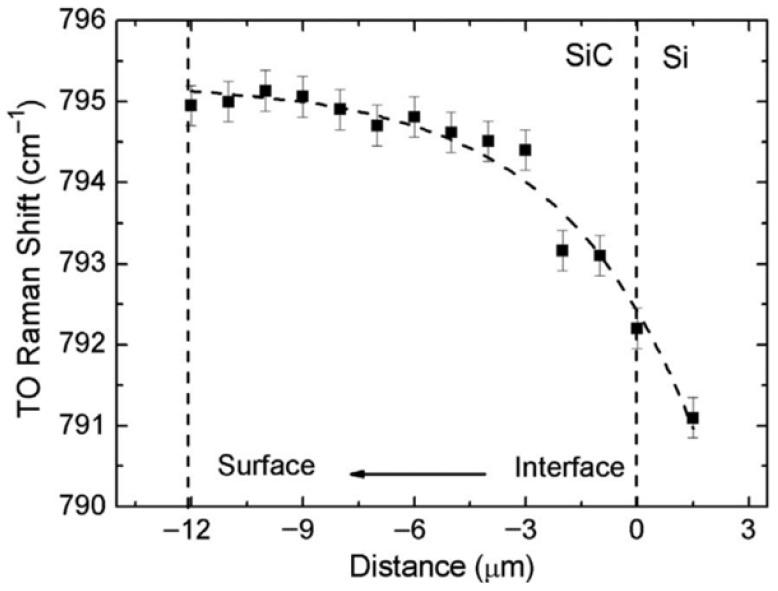
TO Raman shift of the 3C-SiC film as a function of film thickness and exponential fit (dashed line) [20].

The cross-sectional TO Raman shift is reported in Figure 4, showing a clear decrease in stress from the film–substrate interface toward the surface. The 3C-SiC epitaxial film grown on a Si substrate exhibits tensile residual stress, in agreement with the established theoretical framework predicting stress relaxation with increasing film thickness. The stress-free TO Raman peak position for 3C-SiC is approximately 796 cm^−1^. At the film surface, the TO mode indicates a residual tensile stress corresponding to a shift of about 1 cm^−1^, suggesting that the stress is not completely relaxed. As evidenced in Figure 4, the stress evolution with thickness is markedly non-linear and is well described by an exponential fit (dashed line), rather than by a linear approximation. Raman spectroscopy was also employed to assess the crystalline quality of the film. The measured Raman spectrum displays the characteristic features of the cubic 3C-SiC polytype, while the relatively narrow full width at half maximum (FWHM) of the TO mode further confirms the good crystalline quality of the epitaxial layer. Based on the combined TEM and Raman analyses, and supported by FEM modeling, the limitations of the linear stress formulation can be addressed by adopting an exponential approximation for the through-thickness stress distribution, expressed as

(5) $$\sigma_{T}\approx \sigma_{0}\times \exp\left[\sigma_{1}\left(\frac{z}{h/2}\right)\right].$$

In this formulation, the uniform stress component ($\sigma_{0}$) acts as a pre-exponential factor, while the gradient component ($\sigma_{1}$) is incorporated in the exponential term. The coordinate system is defined with its origin at the film midplane; therefore, the thickness coordinate spans the range zϵ[−h/2,h/2].

### 3.3. Defect-Dependent Reduction in Young’s Modulus

The high density of extended defects not only influences the stress state but also degrades the mechanical properties of the material. Anzalone et al. [47] demonstrated that an increased defect density leads to a significant reduction in the effective Young’s modulus of the epitaxial film, with this effect being particularly pronounced in thinner layers. For ideal beams with a rectangular cross section, the mechanical behavior is well described by Young’s modulus, the material density, and the geometric dimensions of the structure, namely length, thickness, and width. Based on these parameters, the resonance frequency of cantilever or bridge structures [47] can be estimated as

(6) $$f=\frac{\lambda_{n}^{2}}{2\pi \sqrt{12}}\frac{t}{L^{2}}\sqrt{\frac{E}{\rho }},$$

where $\lambda_{n}$ is a constant dependent upon the vibrational mode (1.857 and 4.684 for the first and second mode, respectively), t is the cantilever thickness, L is the length, E is Young’s modulus, and ρ is the material density, ρ(3C−SiC)=3210 kg/m^3^.

**Figure 5:**
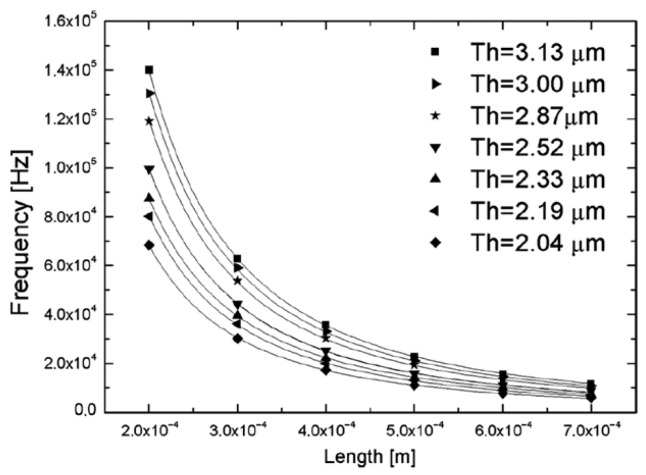
3C-SiC resonant frequencies as function of cantilever lengths for different values of thickness (t) [47].

The trend of the resonant frequency as a function of cantilever length for different values of thickness (t) is shown in Figure 5. Varying the cantilever length results in a wide span of resonant frequencies, ranging, for example, from 145.1±0.2 to 11.8±0.2 kHz of 200–700 μm lengths with a thickness of 3.13 μm. To confirm the experimental data the authors impose, as a fitting function, Equation (4), observing that it is the best function to fit the data. The negligible influence of the bending observed in the as-fabricated structures upon the resonant frequency was also observable from the perfect fit of the data from Equation (4). To validate the experimental results, the data were fitted using Equation (4), which was found to provide the best agreement with the measurements. The excellent fit obtained with Equation (4) also indicates that the bending present in the as-fabricated structures has a negligible influence on the resonant frequency.

### 3.4. High Thermal Stress Component

The elevated temperature required for SiC chemical vapor deposition (CVD) contributes substantially to the overall residual stress, as the difference in the Coefficients of Thermal Expansion (CTE) between SiC and Si generates significant thermal stress upon cooling. Advanced techniques like Selective Epitaxial Growth (SEG) and Epitaxial Lateral Overgrowth (ELO) have been explored to mitigate defect density and residual stress [65].

### 3.5. Material Trade-Offs for MEMS Design

The three structural limitations discussed in Section 3.1, Section 3.2, Section 3.3 and Section 3.4 (defect density, residual stress, and defect-dependent modulus reduction) are not independent: they all originate from the same root cause (the lattice and thermal mismatch at the 3C-SiC/Si interface) and they all evolve with film thickness in a correlated manner. Table 5 summarizes the key trade-offs that must be managed in the design of 3C-SiC MEMS resonators. Thicker films approach bulk crystal quality (higher E, lower defect-related losses) but with reduced residual stress and therefore reduced dilution. The optimal film thickness for Q is consequently not zero nor infinite, but lies in the range ∼600–1000 nm for the geometry and stress levels reported [42], as confirmed by the analytical model of Section 6.1.

## 4. Different Mechanical Properties Depending on Material Structure

The mechanical properties of SiC films are not static, but are strongly dependent on the crystalline structure and morphology of the material.

### 4.1. Crystalline Orientation Dependence (Anisotropy)

SiC is an anisotropic material. Properties, including Young’s modulus and stress behavior, vary depending on the crystallographic orientation. 3C-SiC epitaxial films have been studied in different orientations, such as (100) and (111) on silicon, which directly influence defect growth mechanisms, stress levels, and consequently, the strain sensitivity of resonators [66].

### 4.2. Material Phase (Single-Crystal, Polycrystalline, Amorphous)

The material phase significantly influences performance:

- Monocrystalline SiC (e.g., epitaxial 3C-SiC) is preferred for ultra-high Q-factor resonators due to its superior elastic properties.
- Polycrystalline (Poly-SiC) and Amorphous SiC offer more flexible fabrication routes but generally exhibit lower Young’s modulus values and higher intrinsic mechanical losses.
- Composite structures, such as Polysilicon/3C-SiC beam resonators, have been investigated to manipulate the overall residual stress and achieve enhanced strain sensitivity [66].

### 4.3. Impact of Defect Evolution

The density and type of extended defects, which evolve along the growth direction (decreasing with thickness), are the primary factors governing local variations in Young’s modulus and the resulting stress gradient across the film. This behavior strongly depends on the orientation of the silicon substrate on which the 3C-SiC layer is grown. In fact, in a previous work of Calabretta et al. [13], it is shown that while in 3C-SiC (111) the SF density decreases rapidly, in 3C-SiC (100), it decreases more slowly because new SFs nucleate during growth, limiting the overall improvement of the crystal-quality.

## 5. Different Stress Components

Accurate residual stress analysis is critical for reliable MEMS design. The total stress is decoupled into two main components for modeling. Anzalone et al. used a micromachined planar rotating probe and finite element method (FEM) to study these components [20]:

- Uniform Stress ($\sigma_{0}$): This represents the constant stress component across the film thickness. It is related to the substrate, as well as the lattice and thermal mismatch. This component is essential for defining the pre-tension (stiffening) in tensile-stressed resonant structures, which determines their fundamental resonant frequency.
- Gradient Stress ($\sigma_{1}$): This component describes the variation in stress through the film thickness. It is directly correlated with the evolving defect density (which is maximal at the Si/SiC interface and decreases). The stress gradient is responsible for the bending moment and the resulting out-of-plane deflection (warpage) of suspended microstructures like cantilevers.

**Figure 6:**
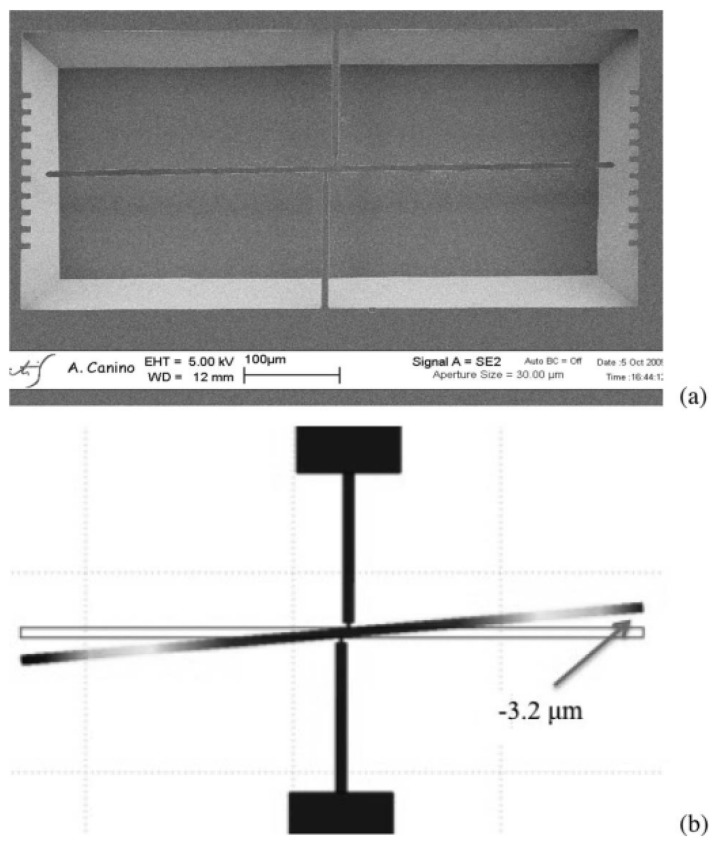
(**a**) SEM image of the micromachined rotating-probe planar deflection y. The distance between the rotation points is 6 μm. (**b**) FEM simulation of the micromachined rotating probe predicting Δx=−3.2μm [20].

**Figure 7:**
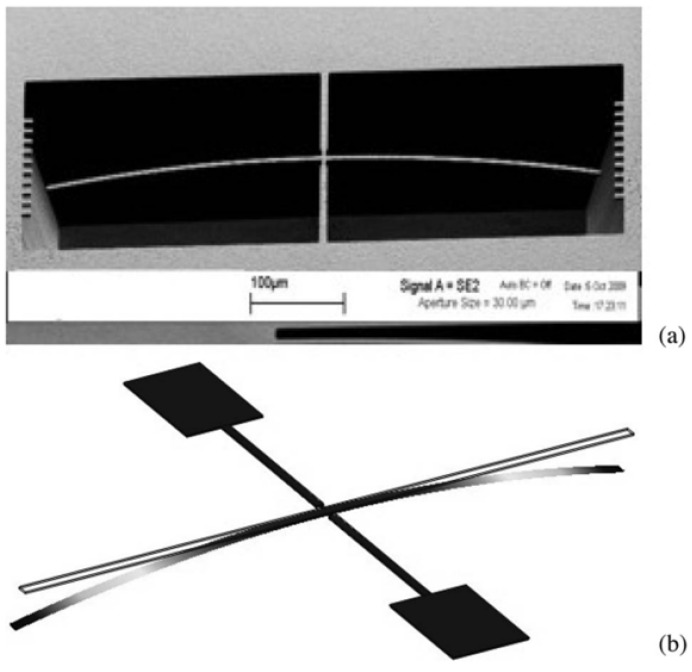
(**a**) A 70° tilted SEM image of the micromachined planar rotating probe with zero displacement ($L_{0}=0$). (**b**) FEM analysis of the z(x) deflection of the central rotating-probe arm due to the gradient residual stress component [20].

The in-plane rotation of the micromachined rotating probe provides direct information on the total average residual stress within the thin film (⟨$\sigma_{T}$⟩), as this motion results from the combined contribution of both the uniform and gradient stress components integrated over the film thickness (Figure 6). In contrast, the out-of-plane deflection of the main arm along the z-axis is primarily governed by the stress gradient across the film thickness and is therefore sensitive only to the gradient component of the residual stress ($\sigma_{1}$). This selective mechanical response allows the rotating probe to effectively decouple the uniform and gradient contributions to the overall residual stress state of the epitaxial film, enabling a more comprehensive and accurate stress characterization. Finite element method (FEM) simulations, in agreement with previously reported studies in the literature, demonstrate that the distance between the turning points of the probe ($L_{0}$) plays a fundamental role in determining the accuracy and sensitivity of the stress measurements. Variations in $L_{0}$ directly influence the planar deflection of the probe tip by altering the mechanical balance between the anchoring beams. As a consequence, different values of $L_{0}$ lead to different levels of stress compensation and measurement sensitivity. Among the possible configurations, the zero-displacement condition ($L_{0}=0$) emerges as the optimal geometry for the reliable extraction of the gradient stress component $\sigma_{1}$ (Figure 7). In this specific configuration, the stresses induced by the two opposing anchorage beams are symmetrically distributed and exactly cancel each other in the planar direction, thereby suppressing spurious contributions from uniform stress. This results in an enhanced sensitivity of the probe to out-of-plane deflection and, consequently, to the stress gradient across the film thickness, improving both the robustness and accuracy of the measurement.

**Figure 8:**
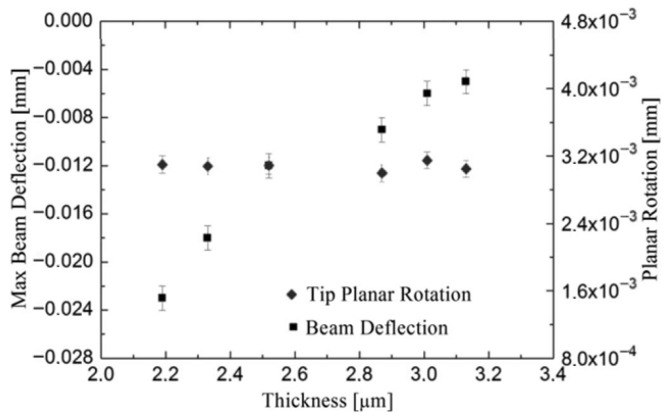
Beam deflection (square) as a function of film thickness for a zero displacement ($L_{0}=0$) rotating probe. Tip planar rotation (rhombus) as a function of the thickness profile [20].

The different thicknesses influence the deflection of the main rotating-probe arm (square), decreasing deflection with increasing thickness (Figure 8). On the other hand, the SEM analysis performed on different thicknesses showed that the planar rotation of the tip (reported in the right zone of the graph) is the same (rhombus) (Figure 8). As predicted from the theory, the tip deflection seems to be influenced only by the distance between the rotating points and not by the film thickness. Therefore, the 3C-SiC film stress $\sigma_{T}$ is constant for different thicknesses. Both n-type and p-type doping have a large effect on the defect reduction [49] and the residual stress [15].

### Exponential Approximation

**Figure 9:**
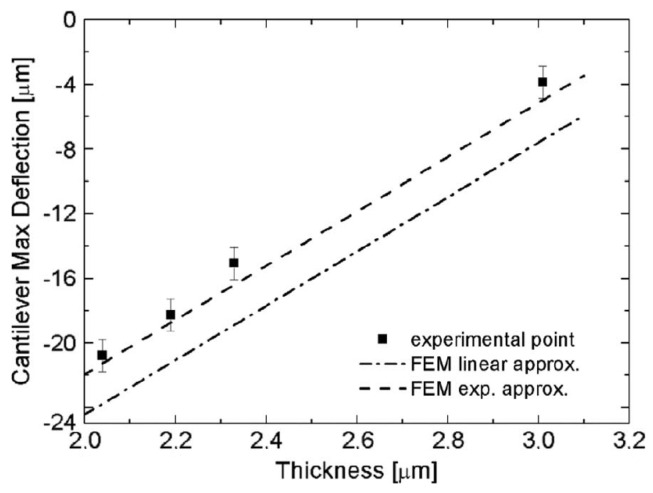
Cantilever maximum deflection as a function of the wafer thickness profile. The graph shows the comparison between the experimental data and two different simulated fits (linear and exponential approximation of the stress function [20]).

Using a simple linear approximation of the stress gradient is incorrect for heteroepitaxial thin films (Figure 9). Instead, an exponential approximation for the stress relationship, correlated with the exponential decay of defect density, provided a significantly better fit to experimental data and resulted in more accurate total residual stress calculation using the finite element method (FEM) [20]. This improved model is vital for predicting the static and dynamic behavior of MEMS devices.

## 6. Quality Factor

The quality factor is a dimensionless quantity used to describe the energy dissipation in a system. In micromechanical resonators, energy losses arise from different mechanisms, including air damping, clamping losses, and thermoelastic dissipation (TED). Air damping is associated with the interaction between the resonator and the surrounding air molecules, and it becomes significant when the device operates at low frequencies and under non-vacuum conditions. Clamping loss refers to the energy that is dissipated through the resonator’s supports and is strongly influenced by the device geometry. Thermoelastic dissipation is a coupled thermomechanical effect in which strain-induced temperature gradients lead to heat flow and, consequently, energy loss. Although a complete analytical model of the quality factor is not yet available, the study by Boisen et al. [67] shows that the mechanical quality factor is determined by both intrinsic and extrinsic damping contributions: the intrinsic factors depend on the material properties and geometry, while the extrinsic ones are mainly related to environmental conditions. A high-quality factor is essential for applications such as high-precision mass sensing, and optimal resonator performance is achieved when the product of frequency and quality factor is maximized. For microbeam structures, the resonance frequency is influenced by the residual stress, elastic characteristics, and the dimensions and shape of the device. By modifying the resonator’s length and width, variations in both the quality factor and resonance frequency can be investigated. A comparison among samples with different sizes shows that doubling the silicon carbide stress results in a 20% increase in resonance frequency, along with an improvement in the quality factor. High residual stress values can be obtained by making the string length much greater than its thickness, which can be achieved through backside etching of the SiC film [63].

### 6.1. Thickness Dependence of the Quality Factor in 3C-SiC MEMS Resonators

Silicon carbide (SiC) micromechanical resonators are increasingly investigated for MEMS applications requiring large resonance frequency, mechanical robustness, and large quality factors (*Q*). In these devices, the achievable *Q* is strongly influenced by film thickness, residual stress, and the relative weight of intrinsic and extrinsic dissipation mechanisms. A comprehensive analytical framework describing the thickness dependence of the quality factor in thin-film 3C-SiC resonators has been developed by Romero et al. [41] and subsequently extended and validated over a wider thickness range [42].

In this model, the total quality factor is expressed as the combination of intrinsic and external loss channels. External dissipation arises from gas damping and clamping (anchor) losses, whereas intrinsic dissipation includes surface losses, volume-related losses associated with defect motion, and thermoelastic damping. For highly stressed trampoline-like and double-clamped beam resonators, thermoelastic damping is predicted to be negligible ($Q_{\mathrm{TED}}∼10^{9}$), allowing the total quality factor to be written as

(7) $$Q^{-1}\left(h\right)=D^{-1}\left(h\right)\;Q_{\mathrm{int}}^{-1}\left(h\right)+Q_{\mathrm{clamp}}^{-1}\left(h\right),$$

where D(h) is the stress-induced dilution factor and $Q_{\mathrm{int}}$ and $Q_{\mathrm{clamp}}$ represent intrinsic and clamping-related dissipation, respectively.

The dilution factor accounts for the enhancement of the effective quality factor due to tensile stress and depends explicitly on the resonator thickness *h*, length *L*, Young’s modulus *E*, and the mean residual stress σ(h). For a double-clamped beam, it can be approximated as

(8) $$D^{-1}\left(h\right)\approx 2\lambda +\pi^{2}\lambda^{2},$$

with

(9) $$\lambda =\frac{h}{L}\sqrt{\frac{E}{12\sigma (h)}}.$$

The thickness-dependent mean stress σ(h) is experimentally extracted from resonance frequency measurements and described by an analytical expression that captures the transition from compressive to tensile stress and the saturation toward a maximum tensile value for increasing thickness. The quantification of D(h), $Q_{int}$ and $Q_{clamp}$ requires identifying the resonance frequency $f_{r,i}$ and the mean stress as a function of thickness σ(h). A possible method to estimate the mean stress is the measurement of the resonance frequency of a released double-clamped beam [68].

**Figure 10:**
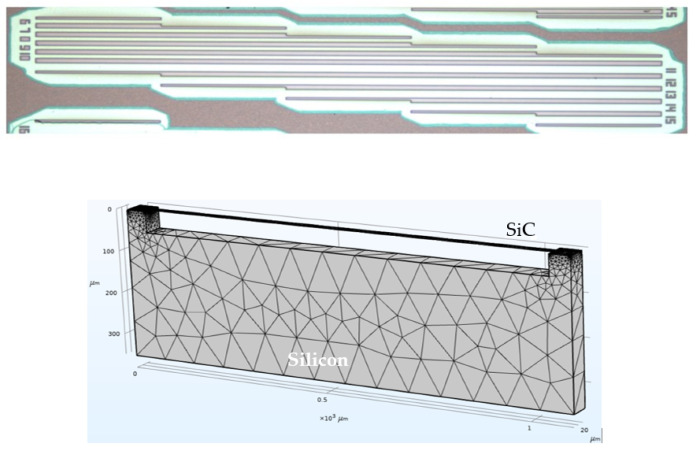
**Top**: double-clamped beam arrays, viewed from the top, with variable length and width [42]; **bottom**: schematic of the fabricated (111) 3C-SiC double-clamped beam resonators, showing the length, width, thickness and anchor configuration.

Clamping losses are modeled as elastic energy radiation into the substrate due to phonon tunneling at the supports [69]. The analytical expression for the clamping-limited quality factor highlights its inverse dependence on film thickness and its sensitivity to residual stress, beam geometry, and substrate elastic properties. Although often neglected in highly stressed resonators, experimental results demonstrate that clamping losses remain a relevant dissipation channel, particularly when intrinsic losses are strongly diluted. The main contribution to the surface losses occurs at the top and the bottom surface of the device due to the larger area than the lateral surface [70], while the volume losses are caused by the defect motion in the volume of the resonator [71]. A key contribution of the model proposed by Romero et al. and further corroborated by a recent work [42] is the description of intrinsic dissipation using a bilayer model which is especially relevant for heteroepitaxial 3C-SiC films grown on silicon substrates (Figure 10).

Surface and volume losses are assumed to act independently in each layer, with surface dissipation scaling inversely with thickness. The total intrinsic quality factor is obtained as a thickness-weighted sum of the contributions from the two layers.

Analysis of experimental data spanning sub-300 nm films [41] up to micrometer-thick resonators [42] reveals a consistent thickness-dependent evolution of the intrinsic quality factor. For thin films, dissipation is dominated by surface losses and by the defect-rich interfacial layer, resulting in relatively low *Q*-values. As the thickness increases, surface-related dissipation becomes progressively less significant, and the intrinsic quality factor converges toward volume-loss-limited values characteristic of the high-quality SiC layer. This behavior confirms that interfacial defects play a central role in limiting *Q* at small thicknesses, while bulk defect motion governs intrinsic dissipation in thicker films.

**Figure 11:**
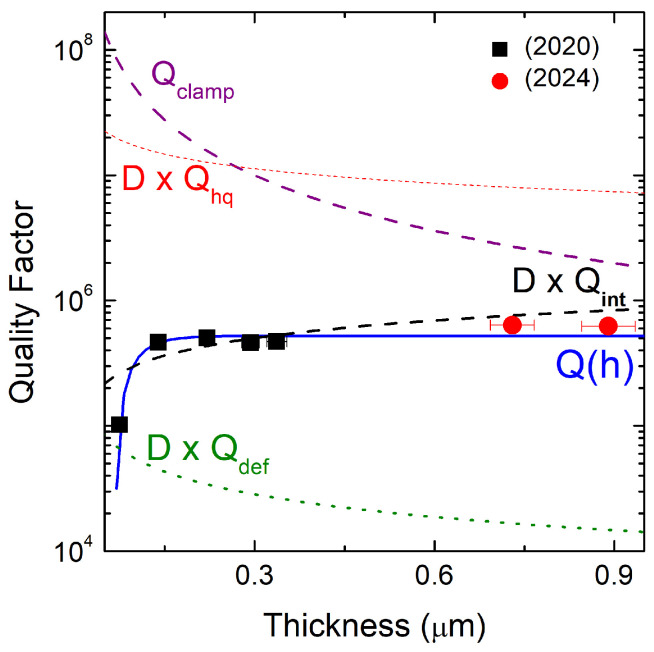
Mean values of the measured Q-factor with different thicknesses. Black squares refer to the total Q-factor for thin film thickness [41]; red circles refer to thicker beams [42], while the dashed blue curve is the theoretical fit of the total quality factor. The dashed red and green curves are, respectively, the limit for the Q-factor when only the high-quality (or rich-defect) layer is considered in the resonator. The dashed purple curve is the external energy dissipation resulting from clamping loss. The dashed black curve is obtained when, for the total Q-factor, it is possible to neglect external energy dissipation via clamping loss, considering only intrinsic energy loss.

By combining the intrinsic bilayer model with the thickness-dependent dilution factor and the analytical formulation of clamping losses, the total quality factor of 3C-SiC double-clamped beams can be accurately reproduced over more than two orders of magnitude in thickness. The model predicts a maximum quality factor of approximately $6\times 10^{5}$ for resonators with a thickness of around 1μm (Figure 11), in excellent agreement with experimental observations. At this thickness, dissipation is primarily governed by intrinsic volume losses in the SiC film, with a secondary but non-negligible contribution from clamping losses.

Overall, the framework developed in Refs. [41,42] provides a robust and physically grounded description of thickness-dependent dissipation in SiC MEMS resonators. By explicitly accounting for stress dilution, anchor losses, and the non-uniform defect distribution inherent to heteroepitaxial 3C-SiC films, this model offers a powerful tool for predicting and optimizing the quality factor of SiC-based MEMS devices, enabling high-resolution sensing and frequency-control applications. These results can be synthesized into a practical design guideline. The total quality factor Q(h) is governed by the competition between two thickness-dependent mechanisms of opposite sign: (i) stress-induced dissipation dilution D(h), which is larger for thinner and more tensile films, and (ii) intrinsic material losses $Q_{int}^{-1}\left(h\right)$, which decrease with thickness as the defect density decays. The product $D\left(h\right)Q_{int}\left(h\right)$ passes through a maximum at an intermediate thickness (approximately 600–1000 nm for the stress and geometry range studied here), which represents the optimal design point for a maximum Q. Below this optimum, surface and defect-dominated losses outweigh the dilution benefit; above it, the reduction in tensile stress progressively erodes the dilution advantage.

### 6.2. Additional Models of Quality Factor in SiC Resonators

Beyond the defect-related dissipation framework recently proposed for (111) 3C-SiC double-clamped beam resonators [42], a broad range of analytical and semi-analytical models have been developed to describe the quality factor of micro- and nanomechanical resonators by isolating specific loss mechanisms and validating them on well-defined material platforms and device geometries. These models provide essential reference points for interpreting dissipation trends in SiC resonators and for contextualizing material-specific formulations within a more general theoretical framework.

A foundational contribution to phenomenological quality factor modeling was provided by Naeli and Brand [72], who performed a systematic experimental and analytical investigation of single-crystal silicon microresonators, including cantilever and clamped–clamped beam geometries, with thicknesses ranging from the micrometer down to the submicrometer scale. The devices were fabricated from monocrystalline silicon and characterized over a wide range of lengths, widths, and aspect ratios, allowing the authors to isolate the dependence of the quality factor on both the geometry and operating environment. Their modeling framework expresses the total quality factor as the inverse sum of independent dissipation channels,

(10) $$\frac{1}{Q_{\mathrm{tot}}}=\frac{1}{Q_{\mathrm{air}}}+\frac{1}{Q_{\mathrm{TED}}}+\frac{1}{Q_{\mathrm{anchor}}},$$

where $Q_{\mathrm{air}}$ accounts for viscous damping due to the surrounding fluid, $Q_{\mathrm{TED}}$ represents thermoelastic damping, and $Q_{\mathrm{anchor}}$ describes energy leakage through the supports. This additive formulation enabled a clear separation between extrinsic and intrinsic loss mechanisms and provided a quantitative tool to identify the dominant dissipation source under different operating conditions. Viscous air damping was shown to dominate the quality factor of slender beams operated at atmospheric pressure, with $Q_{\mathrm{air}}$ strongly dependent on beam width and thickness, consistent with continuum fluid damping models. Under reduced-pressure and vacuum conditions, the contribution of air damping became negligible, revealing the intrinsic loss mechanisms. In this regime, thermoelastic damping emerged as a primary limitation for flexural modes, with the corresponding quality factor described by the classical Zener formulation,

(11) $$Q_{\mathrm{TED}}^{-1}=\frac{E\alpha^{2}T}{\rho c_{p}}\frac{\omega \tau }{1+{\left(\omega \tau \right)}^{2}},$$

where *E* is Young’s modulus, α is the thermal expansion coefficient, *T* is the absolute temperature, ρ is the density, $c_{p}$ is the specific heat, ω is the angular resonance frequency, and τ is the thermal relaxation time across the beam thickness. Experimental measurements confirmed the predicted dependence of TED on thickness and resonance frequency, with thinner beams exhibiting increased dissipation due to shorter thermal diffusion lengths. Support losses were identified as an additional limiting factor in clamped-clamped beams, particularly for shorter devices and higher-order flexural modes. The authors showed that $Q_{\mathrm{anchor}}$ depends strongly on anchor geometry and mode shape, highlighting the role of elastic wave radiation into the substrate as a non-negligible dissipation channel even in otherwise low-loss structures. Measured quality factors ranged from values limited by air damping at atmospheric pressure to significantly higher values under a vacuum, where intrinsic losses dominated, and clear scaling trends with geometry were observed. Although this study was conducted on silicon resonators, the methodology and scaling laws developed by Naeli and Brand [72] provide a general phenomenological framework that is directly applicable to SiC-based devices. In particular, similar dependencies on beam thickness, aspect ratio, and boundary conditions are expected in SiC resonators, while quantitative differences arise from the higher Young’s modulus, thermal conductivity, and reduced intrinsic phonon dissipation of SiC compared to silicon.

Thermoelastic damping (TED) has been extensively modeled and experimentally validated as a fundamental intrinsic loss mechanism in flexural micro- and nanomechanical resonators. The classical theory developed by Zener describes TED as arising from irreversible heat flow driven by cyclic strain gradients that develop between compressed and tensile regions of a vibrating beam, and predicts a characteristic dissipation peak when the thermal diffusion time across the beam thickness matches the oscillation period [73].

Lifshitz and Roukes extended Zener’s treatment by solving the coupled equations of linear thermoelasticity for thin rectangular beams undergoing flexural vibrations, without assuming uniform temperature fields across the cross section [74]. Their analysis, validated on single-crystal silicon cantilevers and doubly clamped beams with submicrometer thicknesses, demonstrated that thermoelastic damping remains relevant down to the nanoscale and that its peak magnitude depends only on the thermodynamic properties of the material, while the position of the dissipation peak is strongly geometry dependent. In particular, they showed that the maximum thermoelastic loss is governed by the relaxation strength $\Delta E=E\alpha^{2}T/c_{p}$ and is largely independent of beam dimensions, whereas the frequency at which peak damping occurs shifts with beam thickness and mode order. Subsequent experimental investigations by Yang et al. [75] further confirmed the strong dependence of TED on beam thickness, temperature, and vibration mode shape through systematic measurements on single-crystal silicon cantilevers. Their results demonstrated good quantitative agreement with thermoelastic models and highlighted that TED sets a fundamental upper bound on the achievable quality factor in low-stress crystalline resonators operating in a vacuum. Collectively, these studies establish thermoelastic damping as an intrinsic and unavoidable loss mechanism in flexural resonators. In high-stress thin films and heteroepitaxial materials, such as SiC-based resonators, the impact of TED can be partially mitigated by dissipation dilution effects, whereby tensile stress increases the stored elastic energy without a corresponding increase in intrinsic material loss. As a result, while the underlying thermoelastic loss mechanisms remain governed by the same physical principles identified in silicon resonators, the observable quality factor in SiC devices can significantly exceed the intrinsic thermoelastic limit predicted for unstressed structures. While the aforementioned models establish fundamental limits and geometry-dependent scaling laws for intrinsic and extrinsic dissipation, their relevance to SiC-based devices has been further clarified through experimental investigations explicitly focused on SiC resonators, where material properties, actuation schemes, and boundary conditions introduce additional constraints on the achievable quality factor. Several experimental studies have specifically addressed dissipation mechanisms in SiC resonators, providing essential benchmarks for quality factor modeling and validating the applicability of classical loss frameworks to wide-bandgap materials. Chang and Zorman [76] investigated lateral-mode resonators fabricated from both single-crystal and polycrystalline 3C-SiC, systematically analyzing the dependence of the quality factor on ambient pressure, temperature, and electrostatic bias conditions. Their measurements showed that gas damping dominates at atmospheric pressure, while operation below the critical pressure leads to a marked increase in *Q*, in agreement with rarefaction-based viscous damping models. Notably, the weak temperature dependence of the measured quality factor over moderate temperature ranges suggested that intrinsic material losses, rather than thermoelastic or surface-related effects, dominate dissipation in these lateral-mode SiC resonators.

Electrothermally actuated SiC resonators were studied in detail by Mastropaolo et al. [77], who fabricated single- and double-clamped beams as well as disk resonators from epitaxial 3C-SiC layers with integrated metallic heaters. Their experimental results highlighted a strong dependence of the quality factor on resonator geometry, with disk-shaped resonators exhibiting quality factors up to ∼$2.3\times 10^{4}$, significantly exceeding those measured in flexural beam structures of comparable dimensions. Although the primary focus of the work was on electrothermal actuation and frequency tuning, the reported *Q*-values indirectly confirmed that anchor losses and thermoelastic dissipation play a dominant role in flexural SiC resonators, whereas bulk-like modes benefit from improved energy confinement. At higher resonance frequencies, phonon-mediated intrinsic losses are more accurately described by the Akhiezer damping model. Akhiezer’s theory attributes sound absorption to the strain-induced modulation of the thermal phonon population and predicts maximum dissipation when the acoustic frequency approaches the inverse phonon relaxation time [78]. Experimental validation in bulk and single-crystal materials confirmed the relevance of this mechanism over a wide temperature and frequency range [79]. More recent studies on bulk acoustic and Lamé-mode resonators have shown that Akhiezer damping can impose an upper bound on the achievable f×Q product.

Support or anchor losses represent a major extrinsic dissipation channel, particularly in clamped-clamped beam and bridge-type geometries. Analytical models developed by Hao et al. describe support losses as elastic wave radiation into the substrate, with dissipation strongly dependent on anchor geometry, mode shape, and substrate properties [80]. These predictions were corroborated by experimental studies showing that anchor losses can dominate the total quality factor even in resonators with intrinsically low material damping. To mitigate this effect, phononic crystal (PnC) engineering has been introduced as an effective strategy to suppress elastic energy leakage. Mohammadi et al. demonstrated high-Q silicon resonators embedded in two-dimensional phononic crystal slabs, achieving quality factors exceeding $10^{6}$ by opening acoustic bandgaps around the resonance frequency [81]. Similar approaches have been successfully applied to SiC-based bulk acoustic and composite resonators, where acoustic confinement leads to substantial quality factor enhancement [82].

As resonator dimensions are further reduced, surface-related dissipation mechanisms become increasingly dominant. Experimental studies on nanomechanical resonators revealed that the quality factor scales inversely with the surface-to-volume ratio, providing strong evidence that surface and interface losses can outweigh bulk material damping [83]. Villanueva and Schmid demonstrated that surface loss is a ubiquitous limiting mechanism even in high-quality crystalline resonators, and proposed two-level system (TLS) models to describe strain-coupled surface defects [70]. These effects are particularly relevant for heteroepitaxial SiC devices, where native oxides, interfacial defects, and fabrication-induced damage can significantly contribute to energy dissipation.

Finally, in bulk-mode and optomechanical resonator geometries, additional modeling efforts have focused on the interplay between mechanical confinement, radiation losses, and surface scattering. Ultra-high-Q microdisk and nanobeam resonators fabricated from single-crystal materials have demonstrated mechanical quality factors exceeding $10^{3}$, with modeling indicating that surface roughness and boundary design often dominate the residual dissipation [84,85].

In this broader context, the various analytical and semi-analytical models developed in the literature provide a comprehensive framework for interpreting the quality factor of SiC resonators and for benchmarking advanced, material-specific formulations (Table 6). Together, they underscore that achieving high Q in SiC devices requires the simultaneous mitigation of thermoelastic, phonon-mediated, anchor-related, and surface-dominated loss mechanisms through careful material selection and resonator engineering.

## 7. Strain Sensitivity

### 7.1. Anisotropic Damping

Before presenting the viscoelastic framework and the FEM results, it is important to explain why anisotropic damping occupies a prominent role in a general review of 3C-SiC MEMS, rather than being treated as a specialist topic. Three reasons motivate this choice. First, isotropic scalar loss-factor models are demonstrably insufficient for crystalline (111) 3C-SiC resonators. As shown in Ref. [86], isotropic simulations systematically overestimate the quality factor for films thicker than approximately 600 nm. This is not a quantitative refinement but a qualitative failure: the isotropic model predicts the wrong trend with thickness. Since film thickness is the primary design variable in heteroepitaxial 3C-SiC, a damping model that fails in this regime is of limited practical utility. Second, the directional nature of the loss mechanisms is not arbitrary but is a direct consequence of the crystal structure and defect topology of the material. Stacking faults and partial dislocations in (111) 3C-SiC propagate preferentially along 111 planes and couple strongly to shear deformation modes. This structural anisotropy is encoded in the off-diagonal terms of the rotated stiffness tensor C(111) (Section 2.3) and must be reflected in the damping formulation. A Voigt-based 6 × 6 loss-factor matrix is therefore not an arbitrary mathematical generalization but the physically natural description of dissipation in this material. Third, accurate Q-factor prediction is a prerequisite for reliable sensor design. For the geophysical monitoring and harsh-environment applications discussed in Section 8, the minimum detectable signal is directly controlled by the resonance linewidth, which depends on Q. Errors of 5–20% in Q prediction-typical of isotropic models for thick 3C-SiC films-translate into comparable errors in noise value estimation and sensor resolution. An anisotropic model is therefore not merely academically interesting but practically necessary.

In 3C-SiC-based MEMS resonators, strain sensitivity quantifies how much the resonance frequency changes with an applied mechanical strain. To first order, the strain sensitivity can be defined as

(12) $$S_{\varepsilon }\;=\;\frac{\partial f}{\partial \varepsilon }\;(\mathrm{or},\;\mathrm{in}\;\mathrm{relative}\;\mathrm{form})\;S_{\varepsilon }^{\mathrm{rel}}\;=\;\frac{1}{f}\;\frac{\partial f}{\partial \varepsilon },$$

where *f* is the resonance frequency and ε is the relevant strain component.

As discussed in the previous paragraphs, the resolution of a resonant sensor depends not only on $S_{\varepsilon }$ but also on frequency noise. Therefore, the high-quality factor (Q-factor) typically achievable in 3C-SiC is a key enabler: a higher Q narrows the resonance linewidth and reduces the equivalent frequency noise, improving the minimum detectable strain [60]. In practice, device optimization often targets maximizing the product f×Q, which is widely used as a compact performance metric for high-performance MEMS resonators [2].

The frequency response and strain sensitivity are strongly influenced by:

- Elastic properties and nominal frequency: The high Young’s modulus of 3C-SiC enables larger resonance frequencies for a given geometry, increasing the information content of the measurement (both in terms of *f* and the f×Q product) [24].
- Defects and thickness dependence: The density of extended defects (SF/TD) and their evolution during growth affect the effective elastic modulus, residual stress, and internal losses; thickness thus becomes a true design parameter rather than a purely technological constraint [43].
- Stress components: Decomposing the residual stress into a uniform stress $\sigma_{0}$ (a mean tensile prestress that stiffens the structure and sets *f*) and a stress gradient $\sigma_{1}$ (responsible for curvature/warpage and local stress variations) clarifies why nominally identical devices can exhibit different responses [20].
- Dissipation mechanisms and Q-factor: Expressing *Q* as the combination of multiple loss channels (fluid damping, thermoelastic damping, anchor losses, surface/material losses, etc.) provides the framework to interpret measurements and identify which mechanism limits the resolution [67].

An important point is the possibility of engineering the residual stress using composite structures (e.g., Poly-Si/3C-SiC) to increase sensitivity: by reducing the mean tensile prestress of the beam, strain sensitivities up to about 1070 Hz/μ strain have been reported for specific composite geometries [66]. This highlights a practical design guideline: sensitivity depends on geometrical parameters, but also on the balance between prestress, stiffness, and the modal strain distribution.

To interpret *Q* properly, internal dissipation must be modeled. Many works adopt an isotropic loss factor (a scalar η equal in every direction); however, in crystalline 3C-SiC, dissipation can be direction-dependent and can vary with the mix of stress states (tension vs. shear) activated by a given vibration mode. This anisotropy is reflected in:

- Mode- and thickness-dependent Q: Different modes and geometries partition elastic energy differently between shear and normal components, leading to systematic variations in *Q*;
- Different tensile vs. compressive sensitivity: If dissipation depends on the stress state, the frequency response under loads of the opposite sign may not be perfectly symmetric (tensile vs. compressive sensitivity) [28,45].

Strain sensitivity must therefore be interpreted as the result of: (i) how the stress state modifies the effective stiffness and thus the resonance frequency *f*, and (ii) how well that change can be resolved, i.e., how “clean” (narrow) the resonance peak is (which depends on *Q*). A more accurate damping model thus improves the prediction of usable sensitivity, not just the nominal frequency.

### 7.2. Hysteretic Viscoelastic Model and Voigt Notation

Under harmonic excitation, dissipation is introduced via a complex modulus (or, in the anisotropic case, a complex stiffness). In one dimension, a typical form is

(13) $$E^{*}\;=\;E^{\prime }+iE^{\prime \prime }\;=\;E\left(1+i\eta \right),$$

where $\eta =E^{\prime \prime }/E^{\prime }$ is the loss factor (often assumed quasi frequency-independent in the hysteretic model) [2]. In three dimensions, Voigt notation is adopted:

(14) $$\sigma ={\left[\begin{array}{cccccc} \sigma_{11} & \sigma_{22} & \sigma_{33} & \sigma_{23} & \sigma_{13} & \sigma_{12} \end{array}\right]}^{T},\;\varepsilon ={\left[\begin{array}{cccccc} \varepsilon_{11} & \varepsilon_{22} & \varepsilon_{33} & \gamma_{23} & \gamma_{13} & \gamma_{12} \end{array}\right]}^{T},$$

with $\gamma_{ij}=2\varepsilon_{ij}$ for the engineering shear strains. The complex stiffness is then written as

(15) $$C^{*}\;=\;C^{\prime }\;\left(I+i\;\eta \right),$$

where *C’* is the real (elastic) stiffness matrix (rotated consistently with the crystallographic orientation, e.g., (111)) [28] and η is:

- Isotropic: η=ηI (a scalar);
- Anisotropic: η is a symmetric 6×6 matrix, with entries $\eta_{IJ}$ weighting specific stress–strain couplings [45].

Within the hysteretic formalism, modal eigenfrequencies become complex:

(16) $${\tilde{\omega }}_{k}=\omega_{k}\;\left(1+i\delta_{k}\right),$$

where $\delta_{k}$ is a dimensionless modal damping (loss) parameter for mode *k*. In the complex-frequency formulation, it equals the ratio between the imaginary and real parts of the modal angular frequency, $\delta_{k}=ℑ\left({\tilde{\omega }}_{k}\right)/ℜ\left({\tilde{\omega }}_{k}\right)$. For light damping, $\delta_{k}$ is directly related to the fraction of energy dissipated per cycle and yields the well-known approximation $Q_{k}≃1/\left(2\delta_{k}\right)$ [73,74].

Thus, for small $\delta_{k}$, one obtains

(17) $$Q_{k,\mathrm{sim}}\approx \frac{1}{2\delta_{k}}\approx \frac{1}{\eta_{\mathrm{eff},k}}.$$

This relation highlights why an inaccurate estimate of η (e.g., forcing isotropy when losses are actually dominated by shear in certain geometries) translates into errors in *Q* and therefore in predicted performance and resolution.

### 7.3. FEM Case Study: Double-Clamped Beams in (111) 3C-SiC

To make the model usable for design, a FEM analysis (e.g., in COMSOL) is applied to real double-clamped beams, including:

- Geometry and boundary conditions: A 3D beam with a rectangular cross-section, clamped at both ends, and free surfaces in the released region; when needed, inclusion of anchor regions and portions of the substrate to capture stress transfer.
- Material: 3C-SiC modeled as a cubic crystal rotated along (111); effective parameters (elastic modulus, residual stress) assigned on a wafer-by-wafer basis [28].
- Damping implementation of both isotropic and anisotropic damping via the matrix [74] in Equation (15).

**Figure 12:**
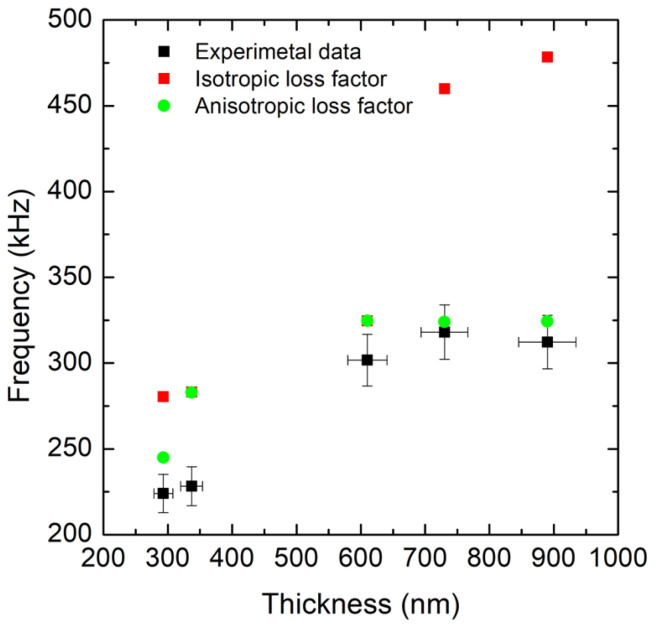
Red trend COMSOL simulations considering the isotropic loss factor in the eigenfrequency analysis. Green circles: COMSOL simulations considering the anisotropic loss factor in the eigenfrequency analysis. Black trend: experimental data. A mesh convergence study was performed, confirming that the selected mesh parameters yield stable results with less than 1% variation in the first eigenfrequency. (Error bands: 5% for experimental resonance frequency and thickness [86].)

In the considered dataset, all beams have nominal length *L* = 1000 μm, while thickness ranges from ∼293 nm to ∼890 nm, with measured frequencies in the ∼230 kH–325 kH interval. In parallel, the effective elastic modulus and residual prestress vary significantly across wafers, reflecting microstructural thickness dependence (Figure 12).

### 7.4. Limitations of the Anisotropic Damping Model

Despite its advantages over the isotropic formulation, the anisotropic loss-factor model presented in this review has several limitations that should be acknowledged. (i) Phenomenological nature. The calibrated $\eta_{IJ}$ matrices are phenomenological descriptors: they reproduce the observed Q-factor trends but do not explicitly separate the physical contributions of thermoelastic damping, anchor (clamping) losses, and surface losses. While order-of-magnitude estimates confirm that thermoelastic damping is negligible in the studied regime ($Q_{TED}>10^{8}$), at least two orders of magnitude above the measured values, anchor losses and surface losses are implicitly embedded in the fitted matrices and cannot be individually quantified from the present dataset. (ii) Absence of a microstructural link. The model provides no direct quantitative relation between the entries of $\eta_{IJ}$ and measurable microstructural quantities such as stacking-fault area density, threading dislocation density, or twin boundary spacing. Establishing such a link would require combined TEM-based defect quantification and frequency-dependent mechanical testing, and is the subject of ongoing work. (iii) Underdetermination risk. Table 3 reports a single representative Q-factor per wafer for compactness, but the calibration procedure uses all experimentally resolved flexural modes (typically 3–5 per wafer in the 200–350 kHz window). Even so, for wafers where fewer modes are resolvable, the number of experimental constraints may approach the number of independent $\eta_{IJ}$ entries (5–6 after symmetry reduction). Multi-mode measurements across a wider frequency range would further constrain the tensor and reduce fitting ambiguity. (iv) Frequency-independence assumption. The hysteretic (frequency-independent) model is appropriate for the 200–350 kHz range studied here, where the Q-values show negligible frequency dependence. This assumption may break down at much higher frequencies (above a few MHz) where relaxation processes may become relevant, or at cryogenic temperatures, where different dissipation mechanisms dominate. (v) Geometric restriction. All calibration data in this work derive from double-clamped beam resonators of fixed length (L = 1000 μm). The applicability of the fitted loss-factor matrices to other geometries (cantilevers, ring resonators, drumhead membranes) has not been experimentally verified and should be tested before using the matrices for cross-geometry predictions.

### 7.5. Loss-Tensor Calibration and Isotropic vs. Anisotropic Comparison

**Figure 13:**
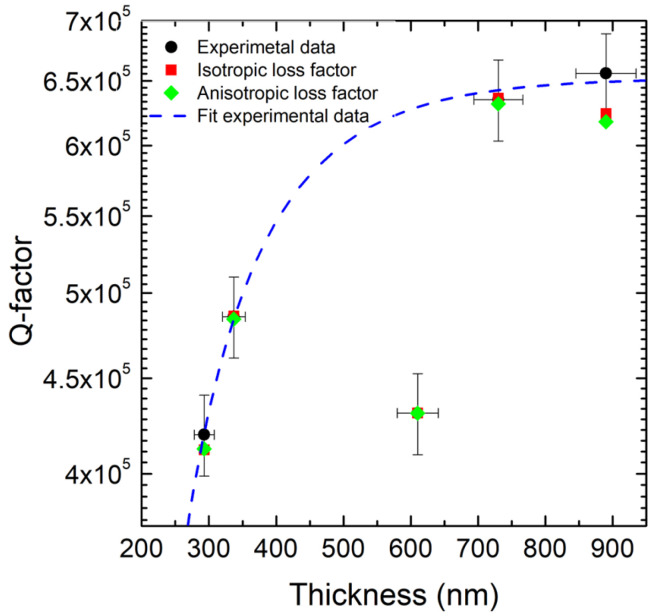
Plot of the Q-factor as a function of material thickness (nm) [86]. The data are shown for three different cases: experimental results (black), simulation with an isotropic loss factor (red), and simulation with an anisotropic loss factor (green). The blue dashed line represents the theoretical fit to the experimental data [41].

To move from a qualitative to a predictive model, the anisotropic matrix η can be calibrated starting from a reference matrix compatible with cubic symmetry and optimizing its entries (within physically plausible ranges) to minimize the error on experimental Q-factors:

(18) $$\Phi =\sum_{k}{\left(Q_{k,\mathrm{sim}}-Q_{k,\exp}\right)}^{2},$$

where the sum runs over the measured modes. This procedure yields a family of fitted η matrices [86] (one per wafer) that:

1. Systematically improves *Q* prediction compared with the isotropic model, with a more pronounced advantage for thicker films (in the analyzed dataset, above a few hundred nm) (Figure 13);
2. Reproduces frequency trends, whereas a scalar isotropic η may overestimate the frequency under certain conditions;
3. Enables a compact quantification of the overall damping magnitude (e.g., via the Frobenius norm of η), which shows a negative correlation with frequency: higher overall dissipation ⇒ lower frequencies.

The Voigt-form analysis also helps interpret which components dominate:

- Thin films: Shear-related components (associated with $\gamma_{23},\gamma_{13},\gamma_{12}$) are often more relevant, especially near the anchors where stress states are more complex [43,45];
- Thick films: Normal components (associated with $\varepsilon_{11},\varepsilon_{22},\varepsilon_{33}$) become more prominent, with a redistribution of losses and a different modal “signature” in terms of *Q* and frequency [43].

This thickness-driven transition is consistent with the idea that changing geometry changes the fraction of elastic energy stored in shear vs. normal deformation and therefore the fraction of energy dissipated per cycle. Practically, anisotropic damping is not a minor detail: it provides a direct route to connect the microstructure, stress state, and dynamic performance.

In the considered measurement set, sensitivity under tensile loads is systematically higher than under compressive loads, indicating a response that is not perfectly symmetric; moreover, sensitivity varies with geometry and thickness [86]:

1. Sensitivity depends on the effective-stiffness change induced by the load (stress engineering and modal distribution);
2. Resolution depends on *Q* and thus on the activated dissipation mechanisms (shear vs. tension, anchor losses, TED, etc.);
3. Microstructure (defects and their thickness evolution) simultaneously affects *E*, $\sigma_{0}$, $\sigma_{1}$, and internal losses, producing trends that cannot be captured by a single isotropic model.

Device performance is optimized by maximizing the product f×Q to reduce frequency noise, enhancing the sensitivity $S_{\varepsilon }$ through geometric and stress engineering (including composite structures) without inducing excessive prestress, and employing anisotropic models based on the Voigt matrix for accurate prediction of *Q*, resonant frequencies, and directional load responses. It is important to distinguish between the formalism and its numerical content. The Voigt-based complex stiffness formulation (Equations (13)–(15)) is a standard result of anisotropic viscoelasticity and is not specific to 3C-SiC. It applies to any crystalline material for which the damping tensor is non-isotropic, including (100)-oriented 3C-SiC, 4H-SiC, GaN, diamond, and Si when operated in orientations that induce shear–normal coupling. The rotation of the stiffness tensor and the parameterization of $\eta_{IJ}$ via Onsager-symmetric matrices are standard procedures of continuum mechanics and are directly portable to other material systems with minimal adaptation. What is material- and orientation-specific is the numerical content of the fitted $\eta_{IJ}$ matrices: the relative magnitude of diagonal (normal-strain) vs. off-diagonal (shear-strain) components, and their evolution with thickness, reflect the particular defect topology and residual stress state of (111) 3C-SiC on Si. These values cannot be transferred directly to other materials or orientations, but the framework for obtaining them, combining FEM eigenfrequency analysis with constrained non-linear calibration of the loss tensor, is fully general. We expect that applying the same methodology to (100) 3C-SiC, 4H-SiC cantilevers, or SiN nanomembranes would reveal material-specific anisotropy signatures consistent with their respective defect structures and crystallographic symmetries.

## 8. Applications of SiC MEMS Technology

As anticipated in the introduction, SiC MEMS devices have been developed over the years to exploit the distinctive properties of SiC and achieve some advantage over traditional Si-based MEMS technologies. The possible advantages of using SiC to fabricate MEMS devices can be generally grouped into the following three main categories: (a) better stability of mechanical/electrical properties of SiC at high temperatures (the goal in this case is generally producing devices that can work well at higher temperatures than their silicon counterparts); (b) superior mechanical properties compared to Si (higher stiffness and yield strength) for devices in which these characteristics are important such as shock accelerometers, mechanical sensors for large forces and mechanical resonators; (c) peculiar chemical properties of SiC such as chemical inertness and interface interaction characteristics (MEMS devices for chemically harsh environments and bio-MEMS). In the following, we are going to mention some examples of SiC MEMS applications reported in the literature. Most of them rely on heteroepitaxial 3C-SiC on Si, which is the focus of this review, even though bulk SiC MEMS have also been increasingly investigated in recent years, thanks to the improvement of bulk SiC micromachining technologies.

### 8.1. Pressure Sensors

Many examples of pressure sensors have been reported in the literature using piezoresistive sensing of the deflection of SiC membranes [87,88]. These devices are normally developed for high-temperature pressure sensing and rely on piezoresistive detection of SiC membrane deflection upon application of pressure on it. Since the creation of piezoresistors in a SiC membrane with ion implantation and annealing is easier on substrates that can withstand high-temperature annealing (above the melting temperature of silicon), most of these examples regard 4H- or 6H-SiC polytypes that cannot be grown by heteroepitaxy on Si, for temperature limitations, and are consequently beyond the scope of the present review article. Some exceptions to the conventional piezoresistor arrangement on a membrane, however, exist, such as the 3C-SiC membrane pressure sensor proposed in [89,90], in which a plain, n-type unintentionally doped 3C-SiC membrane heteroepitaxially grown on Si was used as a pressure sensing element by simply patterning metal electrodes on it and measuring the piezoresistive variation in the electrical resistance measured between these electrodes taking place following the pressure-induced deflection of the membrane. In this way, the impossibility of patterning localized piezoresistors on the 3C-SiC membrane is overcome, even though the obtained pressure sensitivity, in terms of gauge factor per unit pressure applied, appears lower than that of bulk SiC devices that adopt the traditional piezoresistive sensing scheme. With a similar readout scheme, the use of 3C-SiC/Si heterostructures has been recently proposed as an effective means to enhance the pressure sensitivity of heteroepitaxial SiC pressure sensors [91], even exploiting optoelectronic effects driven by light illumination of the sensor membrane during operation [92]. Other than piezoresistive sensing, one of the most popular in the field of pressure sensors, different readout strategies also exist, in which the fabrication of diffused resistors on the SiC membrane is not required. These methods are consequently more readily applicable to technologies based on heteroepitaxial 3C-SiC grown on silicon, such as capacitive [93] or optical [94] sensing of the pressure-induced membrane deflection. In all these cases the readout can be regarded as quite independent of the material used to fabricate the membrane, as long as this material is electrically conductive, as it happens for SiC. The mechanical design of the sensor generally assumes the form of a closed membrane whose area and thickness are adapted to the pressure range of interest for the measurements.

### 8.2. Micromechanical Resonators

Micromechanical resonators are vibrating MEMS structures that can be employed for different applications, such as the sensing of various physical variables (strain, mass, charge), and also for different uses like implementing oscillators for clock signal generation or MEMS for RF signal processing. In all of them, the quality (Q) factor of the micromechanical resonator is of fundamental importance for the performance level of the device. When the resonator is used as a sensor relying on the variation in its mechanical resonance frequency generated by the input variables mentioned above, the resolution of the measurement is related to the Q-factor through the stability of the resonator when it is operated in a closed loop. Such stability is better when the Q-factor of the resonator is high, and, consequently, a better resolution of the frequency-based measurement is possible because the instability of the oscillation in a closed loop represents the noise of the measurement. Similar considerations can be made for the clock generators, in which the stability of the oscillator is fundamental for the quality of the clock signal. As widely discussed in the first part of this review, the Q-factor of a micromechanical resonator depends on several variables, including the mechanical properties of the grown material (such as heteroepitaxial 3C-SiC), the geometrical design of the suspended MEMS structure and the vibration eigenmode utilized at resonance. For its mechanical properties, 3C-SiC is a suitable material to fabricate high-Q-factor resonators. Although the reasons behind this conclusion are not entirely clear yet, it is undoubted that using SiC instead of Si, for instance, vibrating resonators with a much higher Q-factor can be obtained, particularly for thin vibrating structures (thickness lower than 1 µm). This effect was experimentally observed by several groups, who obtained Q-factor values close to 1,000,000 in a high vacuum with 3C-SiC flexural resonators realized on relatively standard heteroepitaxial substrates [69]. As already discussed before in this review, some correlation seems to exist between the presence of a tensile residual stress in the layer and the Q-factor, the latter being higher on more tensile films such as those grown on <111>-oriented Si substrates. However, as was recently reported [95], also films grown on <100>-oriented Si substrates permit the obtaining of relatively high Q-factors, much higher than those ever reported for Si flexural resonators. Another feature that makes SiC resonators interesting is the high Young’s Modulus of the material (more than twice that of silicon [55]) that allows operation of the resonators at higher resonance frequencies. This is convenient for some sensing applications, such as gravimetry. Among the most advanced developments of devices based on SiC resonators reported in the literature so far, we can mention strain sensors [8,96,97], RF devices for signal processing [98], chemical mass sensing devices [99], and single-photon sources [100].

### 8.3. Accelerometers

Research on SiC MEMS accelerometers dates back to the early 2000s. They were among the earliest devices investigated using SiC as an alternative structural material to Si. Since a MEMS accelerometer is an inertial device that measures acceleration by means of the inertial force generated on a suspended mass, the readout scheme can be very similar to that of the pressure discussed above. While in the pressure sensor, the deflection of a membrane taking place after the application of a differential pressure on it must be sensed, in an accelerometer, quite analogously, the deflection of the elastic suspensions used to fix the inertial mass to the substrate needs to be detected and converted into a sensing signal. To do this, the piezoresistor sensing scheme described earlier for the pressure sensors can be conveniently applied by patterning the sensing elements on the mass suspensions in such a way that the movement of the mass induced by the applied acceleration can be sensed electrically. In this respect, the problems related to the patterning of diffused piezoresistors on the SiC material are also the same, and for this reason, heteroepitaxial SiC is not often used in these devices, in which bulk SiC is the material of choice [101,102]. For this reason, and for the need to fabricate sometimes large suspended masses, depending on the target acceleration range, innovative etching techniques have recently been applied to the fabrication of SiC accelerometers, such as femtosecond laser deep micromachining [103]. Of course, other possibilities to sense the displacement of the mass than piezoelectric sensing on the suspensions exist and can be exploited in different types of accelerometers, like those based on capacitive or optical readout [104]. In this case, the use of bulk 6H- or 4H-SiC is less important, and heteroepitaxial SiC can be conveniently adopted for the fabrication. In general, the relatively few examples of SiC MEMS accelerometers presented in the literature so far appeared promising for the possibility of operating on very extended temperature ranges (up to 600 °C) and for sensing very high acceleration levels without damage to the device.

### 8.4. MEMS Devices for Other Applications

Although the three applications reported above have been the most frequently explored in the field of SiC MEMS, other types of devices have also been reported in the literature over the years. Thermal MEMS, for instance, rely on the use of released structures to achieve thermal isolation from the substrate and allow localized heating of selected parts of the device by flowing electrical current through them. Since SiC is very stable at high temperature (mechanically and electrically), it has been successfully used to create infrared light emitters exploiting suspended heating resistance that can be brought at very high temperatures [105]. A similar strategy can be adopted to realize SiC MEMS hotplates for chemical sensing purposes [106]. Other recently proposed applications are in the field of acoustic sensors, in which SiC membranes are utilized as sensing diaphragms for sound waves [107] or in the field of bio-MEMS, where SiC is useful for its biocompatibility, in particular for the one related to the growth of neural cells on it [108].

## 9. Conclusions

This review has provided a comprehensive analysis of silicon carbide (SiC) as a material for microelectromechanical systems, highlighting both its advantages and intrinsic limitations compared with conventional silicon-based MEMS. Particular emphasis has been placed on the strong interdependence between material structure, epitaxial growth conditions, defect density, residual stress, and the resulting mechanical properties of micromachined devices. Special attention has been devoted to heteroepitaxial 3C-SiC grown on silicon substrates, which represents a technologically attractive solution for large-scale MEMS fabrication, despite the presence of extended defects and high residual stress. The impact of these growth-induced features on stiffness, strain sensitivity, and energy dissipation mechanisms has been critically analyzed, demonstrating that device performance cannot be accurately described without explicitly accounting for material anisotropy and defect-related losses. An overview of analytical and numerical models for the quality factor of 3C-SiC resonators has been presented, with particular focus on thickness dependence and the role of stress-induced dilution. Numerical simulations performed using COMSOL Multiphysics have shown that the inclusion of anisotropic loss factors is essential to correctly reproduce the experimentally observed behavior, especially for resonators fabricated on (111)-oriented 3C-SiC films. Finally, the relevance of SiC MEMS applications has been discussed, including pressure sensors, micromechanical resonators, accelerometers, and devices for harsh and high-temperature environments. The superior thermal stability, mechanical robustness, and chemical inertness of SiC enable operation in conditions that are inaccessible to conventional silicon MEMS, making SiC a key enabling material for next-generation sensors and actuators. Future research efforts should focus on further improving epitaxial material quality, refining micromachining techniques, and developing advanced multiphysics models to fully exploit the potential of SiC-based MEMS technologies.

## Figures and Tables

**Table 1:** Measured SiC mechanical properties.

Material Type	Hardness (GPa)	Young’s Modulus (GPa)	References
(100) Si	12.46±0.78	172.13±7.76	[25]
Lely platelet 15R-SiC	42.76±1.19	442±16.34	[25]
3C-SiC on (100) Si	31.198±3.7	433±50	[25]
3C-SiC on (111) Si	>50	>500	[25]
Poly-3C-SiC on (100) Si	33.47±3.3	457±50	[25]
3C	496	223	[26]
3C	401	211	[27]
3C		221.9	[28]
3C	700		[29]
3C epi, undoped	694		[30]
3C epi, p-type	474		[30]
3C epi	330		[31]
3C epi	394		[24]
3C epi	422/435		[32]
3C poly	446		[33]
3C poly	710		[34]
3C poly, <100>	384		[35]
3C poly, random	382		[36]
α-SiC	420		[37]
α-SiC poly	448	225	[35]
α-SiC poly (NC203)	450		[38]
4H		225.9	[28]
6H	441		[39]
6H poly	500		[40]

**Table 2:** Residual stress and Young’s modulus values for SiC sample from two identical depositions [24].

Deposition	Sample	Thickness (μm)	E (GPa)	$\sigma_{0}$ (MPa)
1	1	2.75	381	188
1	2	2.50	459	149
1	3	2.20	456	223
1	4	1.60	292	227
2	1	3.50	377	308
2	2	3.25	398	286
2	3	3.10	403	269
2	4	2.10	385	541

**Table 3:** Range of temperature for different typologies of 3C-SiC.

Typology of 3C-SiC	E at RT	TCYM (ppm/K)	Range of T (°C)	Note
Epitaxial (SC)	330±45	−53±2	RT−330	Film on Si [60]
Epitaxial (SC)	430	−46	RT−450	Undoped film [30]
Epitaxial (SC)	390	−39±5.9	−73 to 20	Low temperature [61]
Polycrystalline (SiC)	452–494	−53	RT−500	Undoped film [62]

**Table 4:** Comparison of different characteristics of clamped SiC (111) microstring resonators with different residual stress and geometries [63].

Material	P (mbar)	Length (μm)	Width (μm)	Thickness (nm)	Residual Stress (MPa)	Q
SiC(111)	$2.2\times 10^{-7}$	220	50	255	750	$3\times 10^{5}$
SiC(111)	$2.2\times 10^{-7}$	1000	4	255	750	$8.2\times 10^{5}$
SiC(111)	$10^{-6}$	930	4	255	1500	$\left(2.9\pm 0.4\right)\times 10^{6}$

**Table 5:** Summary of material trade-offs for MEMS design. (In the table, upward and downward arrows indicate increasing and decreasing trends).

Design Parameter	Effect of ↑ Thickness	Effect of ↓ Thickness	Key Trade-Off
Defect density	↓(annihilation)	↑ (interface proximity)	Thick → better crystal quality
Effective Young’s modulus	↑ (approaches bulk)	↓ (defect softening)	Increasing ∼30–60% from 300 nm to 900 nm
Q-factor (total)	Peaks ∼600–1000 nm	Low (surface + defect loss)	Optimum at intermediate thickness
Stress gradient	↓ (more uniform)	↑ (large gradient)	Thicker beams, flatter but less strain-sensitive
Strain sensitivity	↓ (stiffer, less stress change)	↑ large $\frac{\Delta f}{\Delta \epsilon }$	Thin beams, more sensitive but noisier

**Table 6:** Summary of material platforms, resonator configurations, and reported quality factor values in the selected literature.

Material Type	Resonator Configuration	Quality Factor *Q*	Reference
3C-SiC (111)	DC beam (flexural)	$10^{3}$–$10^{4}$	[41,42]
SiN/crystalline systems	Micro- and nanomechanical beams	∼$10^{5}$ (surface-loss limited)	[70]
Single-crystal Si	Cantilever, CC beam	$10^{3}$ (air) to $10^{5}$ (vacuum)	[72]
Single-crystal Si	Cantilever, CC beam	TED-limited (∼$10^{5}$–$10^{6}$)	[73,74,75]
3C-SiC (single- and polycrystal)	Lateral-mode resonator	∼$10^{3}$ (air), >10^4^ (vacuum)	[76]
Epitaxial 3C-SiC	Beam, disk resonator	Up to ∼$2.3\times 10^{4}$	[77]
Single-crystal Si	Phononic crystal slab resonator	>10^6^	[81]
Mo/SiC composite	Bulk acoustic resonator	>$5\times 10^{4}$	[82]
Single-crystal Si	Nanobeam resonator	$10^{4}$–$10^{5}$ (surface-loss limited)	[83]
Single-crystal 4H-SiC	Microdisk	>$10^{3}$	[84]
Single-crystal Si	Optomechanical crystal	$Q∼10^{6}$, $f\times Q∼10^{13}$ Hz	[85]

## Data Availability

No new data were created or analyzed in this study.
